# Visual aids improve diagnostic inferences and metacognitive judgment calibration

**DOI:** 10.3389/fpsyg.2015.00932

**Published:** 2015-07-16

**Authors:** Rocio Garcia-Retamero, Edward T. Cokely, Ulrich Hoffrage

**Affiliations:** ^1^Department of Experimental Psychology, Facultad de Psicología, University of Granada, Granada, Spain,; ^2^Department of Cognitive and Learning Sciences, Michigan Technological University, Houghton, MI, USA; ^3^Max Planck Institute for Human Development, Berlin, Germany; ^4^National Institute for Risk and Resilience, University of Oklahoma, Norman, OK, USA; ^5^Faculty of Business and Economics, University of Lausanne, Lausanne, Switzerland

**Keywords:** visual aids, Bayesian reasoning, natural frequencies, numeracy, risk literacy, medical decision making, diagnostic inferences

## Abstract

Visual aids can improve comprehension of risks associated with medical treatments, screenings, and lifestyles. Do visual aids also help decision makers accurately assess their risk comprehension? That is, do visual aids help them become well calibrated? To address these questions, we investigated the benefits of visual aids displaying numerical information and measured accuracy of self-assessment of diagnostic inferences (i.e., metacognitive judgment calibration) controlling for individual differences in numeracy. Participants included 108 patients who made diagnostic inferences about three medical tests on the basis of information about the sensitivity and false-positive rate of the tests and disease prevalence. Half of the patients received the information in numbers without a visual aid, while the other half received numbers along with a grid representing the numerical information. In the numerical condition, many patients–especially those with low numeracy–misinterpreted the predictive value of the tests and profoundly overestimated the accuracy of their inferences. Metacognitive judgment calibration mediated the relationship between numeracy and accuracy of diagnostic inferences. In contrast, in the visual aid condition, patients at all levels of numeracy showed high-levels of inferential accuracy and metacognitive judgment calibration. Results indicate that accurate metacognitive assessment may explain the beneficial effects of visual aids and numeracy–a result that accords with theory suggesting that metacognition is an essential part of risk literacy. We conclude that well-designed risk communications can inform patients about healthrelevant numerical information while helping them assess the quality of their own risk comprehension.

## Introduction

Visual aids are graphical representations of numerical expressions of probability. They include, among others, icon arrays, bar and line charts, and grids (Paling, 2003; Spiegelhalter et al., 2011). Visual aids provide an effective means of risk communication when they are *transparent* (Garcia-Retamero and Cokely, 2013)—that is, when their elements are well defined and they accurately and clearly represent the relevant risk information by making part-to-whole relationships in the data visually available (Gillan et al., 1998; Ancker et al., 2006; Reyna and Brainerd, 2008; Fischhoff et al., 2012; Trevena et al., 2012).

Transparent visual aids improve comprehension of risks associated with different lifestyles, screenings, and medical treatments, and they promote consideration of beneficial treatments despite side-effects (Feldman-Stewart et al., 2000; Paling, 2003; Waters et al., 2007; Zikmund-Fisher et al., 2008a; Zikmund-Fisher, 2015). Transparent visual aids also increase appropriate risk-avoidance behaviors, they promote healthy behaviors (Garcia-Retamero and Cokely, 2011, 2014a), they reduce errors and biases induced by anecdotal narratives and framed messages (Fagerlin et al., 2005; Schirillo and Stone, 2005; Garcia-Retamero and Galesic, 2009, 2010a; Cox et al., 2010; Garcia-Retamero et al., 2010) and they aid comprehension of complex concepts such as incremental risk (Zikmund-Fisher et al., 2008b). Risk information presented visually is also judged as easier to understand and recall than the same information presented numerically (Feldman-Stewart et al., 2007; Goodyear-Smith et al., 2008; Gaissmaier et al., 2012; Zikmund-Fisher et al., 2014; Okan et al., 2015).

However, not all visual aids are equally effective for all tasks (see Garcia-Retamero and Cokely, 2013, for a review). For instance, bar graphs are useful for comparing data points (Lipkus and Hollands, 1999; Lipkus, 2007; Fischhoff et al., 2012); line graphs are helpful for depicting trends over time; magnifier risk scales (including magnifying lenses) are useful for depicting small numbers (Ancker et al., 2006); icon arrays can be helpful for communicating treatment risk reduction and risk of side effects (Feldman-Stewart et al., 2000; Garcia-Retamero and Galesic, 2009, 2010b; Ancker et al., 2011; Okan et al., 2012); logic trees can be useful for visually depicting argument structure (Mandel, 2014); and grids can help depict large numbers when communicating the predictive value of medical tests (Garcia-Retamero and Hoffrage, 2013).

Grids displaying numerical information graphically have been found to boost the accuracy of perceptions of health-related benefits and risks beyond the effect of other transparent information formats. To illustrate, doctors and patients often have difficulties inferring the predictive value of a medical test from information about the sensitivity and false-positive rate of the test and the prevalence of the disease. In an influential study on how doctors process information about the results of mammography, Eddy (1982) gave 100 doctors the following information: “The probability that a woman has breast cancer is 1%. When a woman has breast cancer, it is not sure that she will have a positive result on the mammography: she has an 80% probability of having a positive result on the mammography. When a woman does not have breast cancer, it is still possible that she will have a positive result on the mammography: she has a 10% probability of having a positive result on the mammography.”

After having read this information, doctors were required to estimate the probability that a woman with a positive mammography actually has breast cancer. Eddy (1982) reported that 95 of 100 doctors estimated this probability to be about 80% (see Gigerenzer, 2013; Ellis et al., 2014, for similar results in patients). If one inserts the numbers presented above into a Bayes’ theorem, however, one gets a value of 8%, which is one order of magnitude smaller.

Gigerenzer and Hoffrage (1995, 1999) showed that communicating information about medical tests in natural frequencies as compared to probabilities improves diagnostic inferences (see also Sedlmeier and Gigerenzer, 2001; Kurzenhäuser and Hoffrage, 2002; Mandel, 2015). Natural frequencies are final tallies in a set of objects or events randomly sampled from the natural environment (Hoffrage et al., 2000, 2002). For the mammography task the statistical information provided in terms of natural frequencies reads: “100 out of every 10,000 women have breast cancer. When a woman has breast cancer, it is not sure that she will have a positive result on the mammography: 80 of every 100 such women will have a positive result on the mammography. When a woman does not have breast cancer, it is still possible that she will have a positive result on the mammography: 990 out of every 9,900 such women will have a positive result on the mammography.”

Even though the effect of numerical format (probabilities vs. natural frequencies) is substantial, performance in the natural frequency condition still leaves room for improvement. A study conducted by Garcia-Retamero and Hoffrage (2013) showed that grids displaying numerical information graphically improved diagnostic inferences in both doctors and their patients beyond the effect of natural frequencies (see also Brase, 2014, for similar results in young adults). The authors showed that these grids not only increased objective accuracy but also increased perceived usefulness of information and decreased perceived task difficulty. The aim of the current research was to extend this literature by investigating whether visual aids also help decision makers accurately assess their risk comprehension (metacognitive judgment calibration). In particular, we followed the method used by Garcia-Retamero and Hoffrage (2013) and investigated whether grids graphically displaying information about the predictive value of medical tests improve self-assessment of diagnostic inferences in patients.

Previous research showed that people can be highly overconfident when assessing the accuracy of their own judgments (Griffin and Brenner, 2004). For example, Dunning et al. (2004) conducted a systematic review of the literature on the topic and concluded that people’s self-views hold only a tenuous to modest relationship with their actual behavior and performance. On average, people say that they are “above average” in skill—a conclusion that defies statistical possibility for symmetric distributions of individuals (however, this conclusion is plausible if the mean and the median of a distribution are not identical; Gigerenzer et al., 2012). People also overestimate the likelihood that they will engage in desirable behaviors and achieve favorable outcomes, they furnish overly optimistic estimates of when they will complete future projects, and they reach judgments with too much confidence.

People tend to be highly overconfident at low levels of accuracy yet relatively well calibrated at higher levels of accuracy—a result that suggests the presence of an “unskilled and unaware effect” (Ehrlinger and Dunning, 2003; Ehrlinger et al., 2008). This result is consistent with research on individuals with low numeracy (i.e., the ability to accurately interpret numerical information about risk; Ancker and Kaufman, 2007; Fagerlin et al., 2007; Reyna et al., 2009; Galesic and Garcia-Retamero, 2010; Cokely et al., 2012; Peters, 2012). This research shows that people with low numeracy are especially inaccurate when evaluating the accuracy of their own judgments, showing overconfidence (Ghazal et al., 2014), and are not able to use risk reduction information to adjust their estimates (Schwartz et al., 1997). Overconfidence mediates, at least in part, the effect of numeracy on judgment accuracy (Ghazal et al., 2014). Thus, people with low numeracy may struggle to grasp numerical concepts that are essential for understanding health-relevant information because they have difficulties assessing the accuracy of their own estimates.

Our hypothesis is that visual aids can improve both accuracy of diagnostic inferences and metacognitive judgment calibration (i.e., how well patients assess the accuracy of these inferences) (*H_1_*). We also hypothesize that visual aids may be especially useful for patients with low numeracy (*H_2_*). Visual aids can increase the likelihood that less numerate patients deliberate on the available risk information, elaborating more on the problem at hand and on their own understanding of the problem (Garcia-Retamero and Cokely, 2013, 2014b). Deliberation tends to be important for risk understanding because it promotes more thorough, complex, and durable information representations (Cokely and Kelley, 2009)—an important component of metacognitive judgment calibration (Thompson et al., 2011). By influencing encoding and representation, visual aids can increase metacognitive judgment calibration, reducing overconfidence. Improvements in metacognitive processes can, in turn, improve the accuracy of inferences (*H_3_*).

## Materials and Methods

### Participants

Participants included 108 patients recruited from four hospitals in the cities of Jaén and Granada (Spain) during treatment consultation. To be eligible for recruitment, patients had to have no previous formal medical training. If they agreed to participate, they were provided with an introductory letter describing the purpose of the study and their questions were answered. Eighty four percent of the patients who had been approached (*n* = 128) agreed to participate in the study. Those who refused mentioned one or more of the following reasons: respondent burden, lack of interest in research, and/or busy schedules. Patients had an average age of 52 years (range 19–76), and 78% were females. Most of the patients (86%) had a high school degree or less, and only 14% had a university education before participating in the study. Twenty-three percent of the patients had a chronic condition (e.g., allergies or diabetes). Patients received €20 for participating in the study and were assigned randomly to one of two groups. Male and female patients were evenly distributed in the groups. The Ethics Committee of the University of Granada approved the methodology, and all patients consented to participation through a consent form at the beginning of the study.

### Materials and Procedure

Patients completed a two-part paper-and-pencil questionnaire. In the first part, they were presented with three tasks involving different diagnostic inferences: inferring breast cancer from a positive mammogram, colon cancer from a positive hemoccult test, and insulin-dependent diabetes from a genetic test. The order of the three tasks was randomized, independently for each patient. Wording and length of the tasks were comparable to the variant of the breast cancer task that we provided in the introduction of the current article. The information about the sensitivity and false-positive rate of the tests and prevalence of the diseases was taken from published studies (Hoffrage and Gigerenzer, 1998; Garcia-Retamero and Hoffrage, 2013) and was reported in natural frequencies (see Table 1). There were no time constraints, but the questionnaire took approximately 15 min to complete.

**TABLE 1:** **Information about prevalence of the diseases, and sensitivity and false-positive rate of the tests**.

**Diagnostic task**	**Base rate**	**Sensitivity**	**False-positive rate**	**Positive predictive value**
Breast cancer	100 of 10,000	80 of 100	990 of 9,900	80 of 1,070
Colon cancer	30 of 10,000	15 of 30	299 of 9,970	15 of 314
Diabetes	50 of 10,000	48 of 50	4,975 of 9,950	48 of 5,023

Note that the false-positive rate is the complement of the specificity.

**FIGURE 1:**
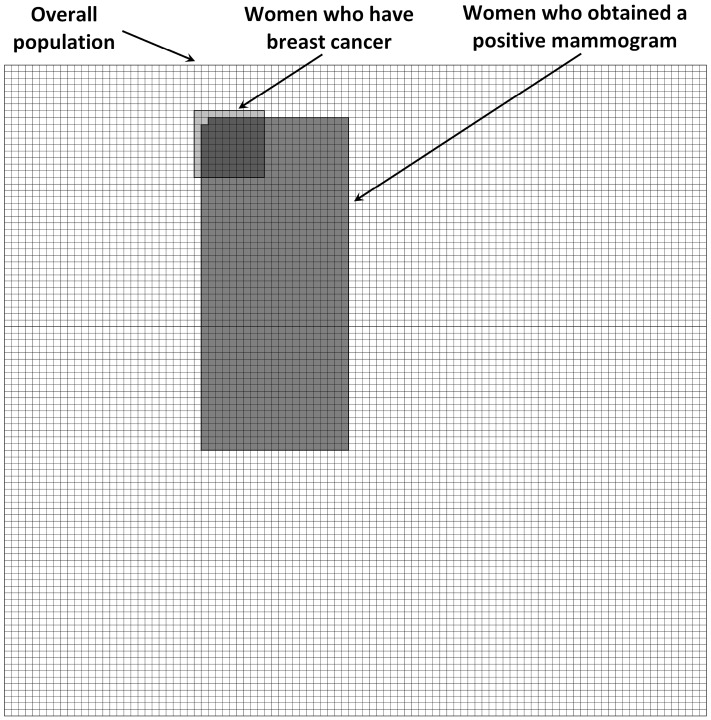
**Visual aid representing the overall number of women at risk, the number of women who have breast cancer, and the number of women who obtained a positive mammogram**.

Half of the patients received the information about the sensitivity and false-positive rate of the tests and prevalence of the diseases in numbers without a visual aid. The other half received numbers along with a grid representing the numerical information. Figure 1 presents the grid that patients received in the mammography task. The visual display represented the number of women who obtained a positive mammogram, the number of women who have breast cancer, and the overall number of women at risk. Women were depicted as squares as previous research has found no differences in effects of arrays with faces compared to more abstract symbols such as squares or circles (Stone et al., 2003; Gaissmaier et al., 2012).

After having received the information about the sensitivity and false-positive rate of the test and the base rate of the disease for a given task, patients made a diagnostic inference. In the breast cancer task, patients were told: “Imagine a representative sample of women who got a positive result on the mammography. Give your best guess: how many of these women do you expect to have breast cancer?” Patients were asked to provide two numbers such as X out of Y (leaving it up to them which denominator to use). After making the three diagnostic inferences, patients estimated accuracy of their diagnostic inferences. In particular, they estimated the number of correct inferences that they thought they had made on a scale ranging from 0 to 3. The second part of the questionnaire included a measure of numerical skills using twelve items taken from Schwartz et al. (1997) and Lipkus et al. (2001; see Cokely et al., 2012, for a review).

### Design and Dependent Variables

We employed a mixed design with one independent variable manipulated experimentally between-groups: information format (numerical only vs. numerical and visual). In addition, we considered one independent variable that was not manipulated experimentally but measured, namely numeracy. We split patients into two groups according to the median of their numeracy scores. The low-numeracy group (*n* = 52) included patients with eight or fewer correct answers, while the high-numeracy group (*n* = 56) included those with nine or more correct answers (see Peters et al., 2006; Garcia-Retamero and Galesic, 2010b; and Garcia-Retamero and Cokely, 2014a, for a similar procedure).

Patients answered questions about the three tasks involving different diagnostic inferences. We used patients’ answers to the questions to determine our three dependent variables. *Objective accuracy* was measured as the percentage of correct inferences in the three tasks. Following Gigerenzer and Hoffrage (1995; see also Hoffrage et al., 2000), a response was considered accurate if it matched the value specified in the last column of Table 1 plus/minus one percentage point. A more liberal criterion than the one that we used in our analyses yielded similar findings to those reported in the results section. *Estimated accuracy* was measured as the estimated percentage of correct inferences in the three tasks. Finally, *metacognitive judgment calibration* was determined for each patient by computing the difference between estimated accuracy and objective accuracy (see Ghazal et al., 2014, for a similar method).

### Analyses

First, we conducted analyses of variance (ANOVAs) to assess the effect of information format and numeracy on objective accuracy, estimated accuracy, and metacognitive judgment calibration (*H_1_* and *H_2_*). Second, we assessed whether metacognitive judgment calibration explains the effect of information format and numeracy on objective accuracy (*H_3_*). In particular, we conducted an analysis of covariance (ANCOVA) to assess the effect of information format and numeracy on objective accuracy after controlling for metacognitive judgment calibration. We also conducted mediational analyses to assess whether the effect of information format and numeracy on objective accuracy was mediated by metacognitive judgment calibration.

Finally, to find additional support of our hypothesis (*H_3_*) and address an alternative explanation of our results, we investigated whether objective accuracy explains the effect of information format and numeracy on metacognitive judgment calibration. In particular, we conducted an ANCOVA to assess the effect of information format and numeracy on metacognitive judgment calibration after controlling for objective accuracy. In addition, we conducted mediational analyses to assess whether the effect of information format and numeracy on metacognitive judgment calibration was mediated by objective accuracy. As this alternative model seems plausible, we compared the size of its indirect effect (i.e., the amount of mediation) with that of the model with metacognitive judgment calibration as a mediator. Numeracy was included as a dichotomous variable in the ANOVAs and ANCOVAs and as a continuous variable in the mediation analyses. We found consistent results in these analyses (for a similar method, see Peters et al., 2006; Garcia-Retamero and Galesic, 2009, 2010b; and Garcia-Retamero and Cokely, 2014a).

## Results

**FIGURE 2:**
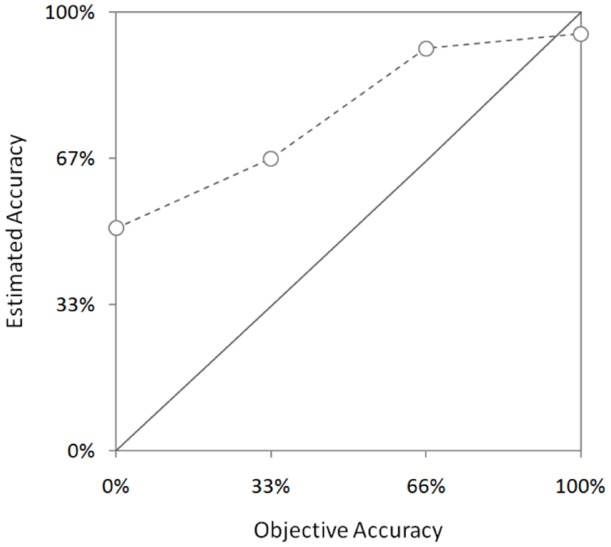
**Estimated accuracy by objective accuracy.** Error bars indicate one standard error of the mean.

*How did patients perform in the diagnostic inference tasks? And how did they think they had performed in the tasks?* The percentage of patients who answered correctly three, two, one, and zero tasks was 24, 19, 18, and 39% respectively. In contrast, 50, 25, 19, and 6% of the patients estimated that they had made three, two, one, and zero correct diagnostic inferences, respectively. Only 34% of the patients were accurate when assessing the accuracy of their inferences (i.e., they were well calibrated); 38% overestimated accuracy in one task (33%); 18% overestimated accuracy in two tasks (67%); 6% overestimated accuracy in three tasks (100%); and 4% underestimated accuracy. Patients who achieved higher levels of accuracy were well calibrated, whereas patient with low levels of accuracy were highly overconfident (see Figure 2).

**FIGURE 3:**
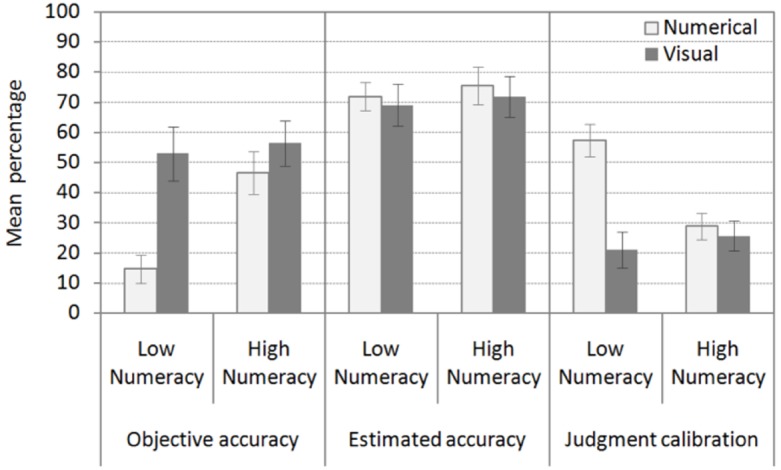
**Objective accuracy, estimated accuracy, and metacognitive judgment calibration across the three diagnostic tasks by information format and numeracy.** Error bars indicate one standard error of the mean.

*Do visual aids and numeracy affect objective accuracy? Are visual aids especially useful for patients with low numeracy?* Patients made more accurate inferences when the information was presented both numerically and visually (55% correct inferences) as compared to numerically only (32%) (*H_1_*). In addition, patients with high numeracy were more accurate (51% correct inferences) as compared to low-numerate patients (35%). Finally, grids displaying numerical information were particularly useful additions for patients with low numeracy (see Figure 3). In contrast, there was only a minor increase in accuracy in patients with high numeracy when they received the additional visual display (*H_2_*). In line with these results, the ANOVA with information format and numeracy as between-subjects factors and objective accuracy across the three tasks as the dependent variable revealed a main effect of information format, *F*_1,104_ = 10.77, *p* = 0.001, ${\text{η}}_{p}^{2}$ = 0.09, and numeracy, *F*_1,104_ = 5.79, *p* = 0.02, ${\text{η}}_{p}^{2}$ = 0.05. The interaction between information format and numeracy was also significant, *F*_1,104_ = 3.82, *p* = 0.05, ${\text{η}}_{p}^{2}$ = 0.04.

*Do visual aids and numeracy affect estimated accuracy and metacognitive judgment calibration? Are visual aids especially useful for patients with low numeracy?* Estimates of accuracy were not influenced by information format or numeracy. On average, patients estimated that 72% of their inferences were correct (see Figure 3). In contrast, both information format and numeracy had an effect on accuracy of estimates (i.e., metacognitive judgment calibration) (*H_1_*). Grids displaying numerical information improved metacognitive judgment calibration in patients with low numeracy. These patients more accurately estimated the accuracy of their own inferences when they received the visual aid. However, the beneficial effect of the visual aid could not be observed in patients with high numeracy (*H_2_*). These patients were relatively well calibrated regardless of information format. In line with these results, the ANOVA with information format and numeracy as between-subjects factors and estimated accuracy of diagnostic inferences across the three tasks as a dependent variable did not reveal any significant results (*F* < 1). In contrast, the ANOVA with information format and numeracy as between-subjects factors and metacognitive judgment calibration as a dependent variable revealed a main effect of information format, *F*_1,104_ = 14.62, *p* = 0.001, ${\text{η}}_{p}^{2}$ = 0.12, and numeracy, *F*_1,104_ = 5.28, *p* = 0.02, ${\text{η}}_{p}^{2}$ = 0.05, and an interaction between information format and numeracy, *F*_1,104_ = 10.22, *p* = 0.002, ${\text{η}}_{p}^{2}$ = 0.09.

**FIGURE 4:**
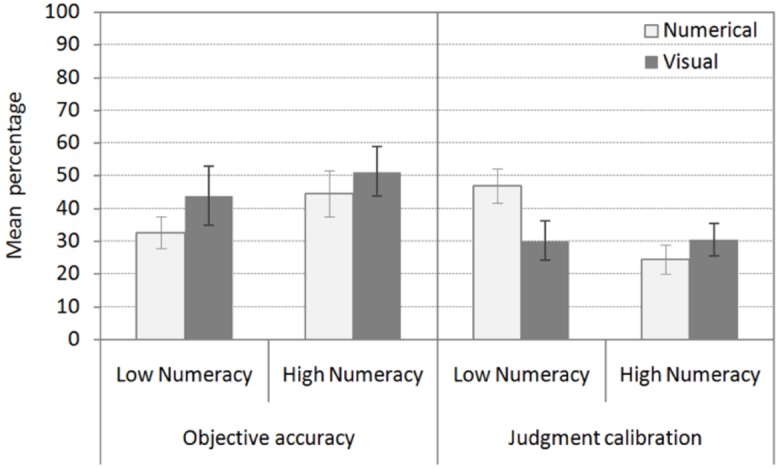
**Objective accuracy across the three diagnostic tasks by information format and numeracy after controlling for the effect of metacognitive judgment calibration.** Metacognitive judgment calibration across the three diagnostic tasks by information format and numeracy after controlling for the effect of objective accuracy. Error bars indicate one standard error of the mean.

*Does metacognitive judgment calibration explain the effect of information format and numeracy on objective accuracy?* Visual aids do not improve objective accuracy in patients with low numeracy when metacognitive judgment calibration has been controlled for statistically (see Figure 4). In line with these results, the ANCOVA with information format and numeracy as between-subjects factors, objective accuracy across the three tasks as the dependent variable, and metacognitive judgment calibration as a covariate only revealed a main effect of metacognitive judgment calibration, *F*_1,103_ = 37.25, *p* = 0.001, ${\text{η}}_{p}^{2}$ = 0.27. The main effect of information format and numeracy and the interaction between information format and numeracy was no longer significant (*F* < 1).

**FIGURE 5:**
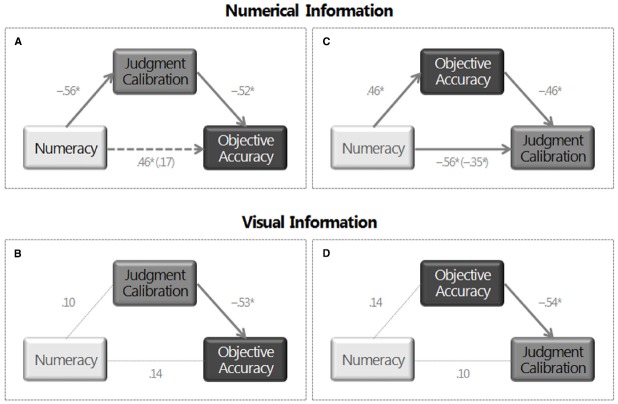
**Path analyses.** Effect of numeracy on objective accuracy and the mediational effect of metacognitive judgment calibration when **(A)** patients received information only in numbers and when **(B)** they received an additional visual display representing the numerical information. Effect of numeracy on metacognitive judgment calibration and the mediational effect of objective accuracy when **(C)** patients received information only in numbers and when **(D)** they received an additional visual display representing the numerical information. Note: Standardized coefficients are shown. *p < 0.05.

To ensure comparability with results in the ANOVA and ANCOVA, in mediational analyses we first modeled objective accuracy when patients received information in numbers and then when they received an additional visual display representing the numerical information. In the numerical condition, regression analyses showed that numeracy influenced both metacognitive judgment calibration, β = –0.56, *t*_53_ = –4.97, *p* = 0.001, and objective accuracy, β = 0.46, *t*_53_ = 3.82, *p* = 0.001, whereby patients who were more numerate more accurately assessed the accuracy of their inferences (i.e., were better calibrated) and made more accurate inferences (see Figure 5A). In addition, metacognitive judgment calibration was related to objective accuracy, β = –0.52, *t*_52_ = –4.04, *p* = 0.001. Patients who more accurately assessed the accuracy of their inferences also made more accurate inferences. When metacognitive judgment calibration was included in the regression analyses, the effect of numeracy on objective accuracy was significantly reduced and was no longer significant, β = 0.17, *t*_52_ = 1.30, *p* = 0.20. The results of the Sobel test indicated that metacognitive judgment calibration mediates the relationship between numeracy and objective accuracy, *z* = 3.135, *p* = 0.001 [Effect = 0.30, 95% CI (0.27,0.33); AIC (Akaike Information Criterion) = 998.80]. When patients received the additional visual aid representing the numerical information, numeracy did not influence metacognitive judgment calibration, β = 0.10, *t*_51_ = 0.70, *p* = 0.49, or objective accuracy, β = 0.14, *t*_51_ = 1.01, *p* = 0.32 (see Figure 5B). As expected, metacognitive judgment calibration was again related to objective accuracy, β = –0.53, *t*_50_ = –4.48, *p* = 0.001.

*Does objective accuracy explain the effect of information format and numeracy on metacognitive judgment calibration?* Visual aids improve metacognitive judgment calibration in patients with low numeracy after objective accuracy has been controlled for statistically (see Figure 4). The ANCOVA with information format and numeracy as between-subjects factors, metacognitive judgment calibration across the three tasks as the dependent variable, and objective accuracy as a covariate revealed a main effect of objective accuracy, *F*_1,103_ = 37.25, *p* = 0.001, ${\text{η}}_{p}^{2}$ = 0.27, and information format, *F*_1,103_ = 5.56, *p* = 0.020, ${\text{η}}_{p}^{2}$ = 0.05, and an interaction between information format and numeracy, *F*_1,103_ = 6.24, *p* = 0.014, ${\text{η}}_{p}^{2}$ = 0.06.

As expected, in the numerical condition, regression analyses showed that objective accuracy was related to metacognitive judgment calibration, β = –0.46, *t*_52_ = –4.04, *p* = 0.001 (see Figure 5C). Patients who made more accurate inferences also more accurately assessed the accuracy of these inferences. When objective accuracy was included in the regression analyses, the effect of numeracy on metacognitive judgment calibration was reduced but it was still significant, β = –0.35, *t*_52_ = –3.12, *p* = 0.003. The results of the Sobel test indicated that objective accuracy mediates the relationship between numeracy and metacognitive judgment calibration, *z* = –2.78, *p* = 0.003. However, the size of the indirect effect [Effect = –0.21, 95% CI (–0.24, –0.18)] was smaller and AIC (AIC = 1057.30) was larger to that of the previous model. These results suggest that the model including objective accuracy as a mediator is a worse model than the model including metacognitive judgment calibration as a mediator.

In line with previous results, when patients received the additional visual aid representing the numerical information, objective accuracy was related to metacognitive judgment calibration, β = –0.54, *t*_50_ = –4.48, *p* = 0.001 (see Figure 5D). In sum, results in ANCOVAs and mediational analyses suggest that metacognitive judgment calibration mediates the effect of numeracy on objective accuracy (*H_3_*) and not the other way around. Thus these analyses suggest that, in the numerical condition, highly numerate patients make more accurate inferences than patients with low numeracy because they more accurately evaluate the accuracy of their own inferences. In contrast, in the visual condition, patients at all levels of numeracy showed similar high-levels of metacognitive judgment calibration and, in turn, high-levels of inferential accuracy.

## Discussion

We investigated patients’ diagnostic inferences about the predictive value of medical tests from information about the sensitivity and false-positive rate of the tests and the prevalence of several diseases. Our results showed that many patients—especially those with low numeracy—made incorrect inferences about the predictive value of the tests and dramatically overestimated the accuracy of these inferences. High overestimates at low levels of accuracy become more calibrated at higher levels of accuracy—a result that suggests the presence of an “unskilled and unaware effect” (see also Ehrlinger and Dunning, 2003; Ehrlinger et al., 2008; Ghazal et al., 2014).

Our results are compatible with previous evidence on the role of numeracy in understanding health-relevant risk communications and medical decision making (Fagerlin et al., 2007; Apter et al., 2008; Reyna et al., 2009; Peters, 2012; Garcia-Retamero and Galesic, 2013; Johnson and Tubau, 2015). Patients with low levels of numeracy have more difficulties interpreting numerical risks of side effects (Gardner et al., 2011), and they are more susceptible to being influenced by the way the health information is framed in problems involving probabilities (Peters et al., 2006; Peters and Levin, 2008; Garcia-Retamero and Galesic, 2010a, 2011; Galesic and Garcia-Retamero, 2011a)—presumably because they are more influenced by non-numerical information (e.g., mood states; Peters et al., 2007; Petrova et al., 2014). Compared to patients with high numeracy, less-numerate patients also tend to overestimate their risk of suffering from several diseases (Davids et al., 2004; Gurmankin et al., 2004), they are less able to use risk reduction information to adjust their risk estimates (e.g., screening data; Schwartz et al., 1997), they tend to overestimate benefits of uncertain treatments (Weinfurt et al., 2003; Garcia-Retamero and Galesic, 2010b), and they have more deficits in understanding the information necessary to follow dietary recommendations (Rothman et al., 2006). Compared to patients with high numeracy, less-numerate patients also tend to search for less information about their disease (Portnoy et al., 2010), and they often choose lower-quality health options (e.g., health insurance plans; Hibbard et al., 2007; Hanoch et al., 2010). As a consequence, they tend to suffer more comorbidity and take more prescribed drugs (Garcia-Retamero et al., 2015). Less-numerate doctors and patients also favor a paternalistic model of medical decision making, in which doctors are dominant and autonomous (Garcia-Retamero et al., 2014), and patients prefer not to participate and instead delegate decision making (Galesic and Garcia-Retamero, 2011b). This is troubling given that the paternalistic model of medical decision making is increasingly being questioned (Kaplan and Frosch, 2005).

Our research suggests a potential explanation of the link between numeracy and understanding of health-relevant quantitative information. Highly numerate patients might make more accurate inferences as compared to patients with low numeracy because they more accurately evaluate the accuracy of their own inferences (i.e., they show better metacognitive judgment calibration). Thus metacognitive judgment calibration might drive, at least in part, the numeracy-to-performance relationship. Previous research suggests that the link between numeracy and superior judgment and decision making might reflect differences in heuristic-based deliberation (e.g., deep elaborative processing; Cokely and Kelley, 2009; Cokely et al., 2012), affective numerical intuition (e.g., precise symbolic number mapping; Peters et al., 2006; Peters, 2012), and meaningful intuitive understanding (e.g., gist-based representation and reasoning; Reyna, 2004; Reyna et al., 2009; see Cokely et al., 2014, for a review). Our research extends this literature suggesting that there is also a tight link between numeracy, metacognition, and understanding of health-relevant numerical information (see Ghazal et al., 2014, for similar results in highly educated samples).

Our results are also compatible with a variety of studies indicating that judgment self-assessment can operate as a domain-general skill that correlates with—but that can also be seen as an independent predictor of—general abilities, personality traits, and cognitive performance (Stankov, 2000; Stankov and Lee, 2008; Schraw, 2010). Overall our results accord with metacognitive theory suggesting that metacognitive judgment calibration tends to be useful because it is instrumental in self-regulation—i.e., the monitoring and control of cognition (Nelson, 1990; Metcalfe and Finn, 2008). Related studies of factors like “feeling of correctness” show that confidence-type judgments predict differences in information search and elaboration. In addition to predicting judgments about the correctness of one’s answer, one’s feeling of correctness tends to be related to “rethinking” times and the likelihood of changing one’s initial answer during reasoning (Thompson et al., 2011). These studies suggest that factors related to how one uses and assesses judgment accuracy may often be essential components determining the extent to which one deliberates during judgment and decision making (Ghazal et al., 2014). For these and other reasons it seems likely that metacognition is an essential component of the ability to understand and make good decisions about risk (i.e., risk literacy; see www.RiskLiteracy.org).

Finally, our results can have important implications for medical practice as they suggest suitable ways to communicate quantitative medical data—especially to patients lacking numerical skills. Our research shows that visual aids improve both objective accuracy and metacognitive judgment calibration, especially in less numerate patients, eliminating differences between this group of patients and the more numerate group. In addition, our research suggests that visual aids increase objective accuracy by improving metacognitive judgment calibration. As we mentioned above, calibration can mediate the relationship between numeracy and superior performance. In the current research, however, this result only holds when patients received numerical information without a visual display. In contrast, metacognitive judgment calibration did not mediate the effect of numeracy on objective accuracy when patients received the additional visual aid representing the numerical information because numeracy was no longer as robustly related to accuracy of inferences. In the visual condition, both patients with low and high numeracy were often well calibrated and, in turn, often made accurate inferences. These results suggest that visual aids might improve risk understanding, at least in part, by improving metacognitive judgment calibration and reducing overestimates of accuracy.

It is also possible that the effect of visual aids on both judgment accuracy and metacognitive judgment calibration follow from the development of better cognitive representations, which, in turn, facilitate reasoning and metacognitive monitoring (see Cosmides and Tooby, 1996; Brase et al., 1998; Brase, 2009). For instance, more cues available in memory can be used to explore essential relationships or to recognize that one has some missing knowledge. This conclusion is compatible with previous research indicating that visual aids help less numerate people identify and infer essential aspects of the risk information (e.g., “gross-level information”; Feldman-Stewart et al., 2000; Zikmund-Fisher et al., 2010). Visual aids also increase the ability of less numerate people to recognize superordinate classes, making part-to-whole relations in the data visually available (Ancker et al., 2006; Reyna and Brainerd, 2008). Moreover, visual aids improve risk comprehension by increasing the likelihood that less numerate people deliberate on the available risk information (Garcia-Retamero and Cokely, 2013, 2014b). By influencing memory encoding and representation, visual aids can also give rise to enduring changes in attitudes and behavioral intentions, which in turn affect behavior and risky decision making (Garcia-Retamero and Cokely, 2011, 2014a, 2015). Thus visual aids can improve judgment and decision making and help promote healthy behavior by improving understanding of health-relevant numerical information, by improving assessments of the accuracy of inferences about this information, and by establishing enduring attitudes and fostering intentions to perform the behavior, which may further promote understanding and self-assessment.

As with any research, our study has some limitations and leaves open several questions for future research. For instance, objective accuracy and metacognitive judgment calibration were correlated as the former was included in the measurement of the latter. To the extent that judgment calibration cannot be defined independently of objective accuracy, these concepts are not independent. So any results in this area need to be benchmarked accordingly. Nevertheless, our analyses showed that information format and numeracy have a significant effect on metacognitive judgment calibration even after objective accuracy has been controlled for statistically.

It is important to mention that our conclusions are based on patients’ diagnostic inferences and estimates when they received information about prevalence of several diseases, and the sensitivity and false-positive rate of the tests in natural frequencies (Hoffrage and Gigerenzer, 1998; Hoffrage et al., 2000, 2002). Future research could investigate these inferences and estimates when the information is reported in other numerical formats (e.g., probabilities). In addition, future research could also investigate whether these inferences and estimates affect behavioral intentions and actual behavior (e.g., whether patients indicate that they would take a medical test depending on the way the information about the test is communicated and if expressed interest exceeds actual uptake). Our sample of patients was older and less educated than the general population in Spain and other countries. Future research could also examine whether visual aids confer similar results in more educated participants (e.g., physicians) in different countries. Finally, future research could investigate whether the general findings hold across different types of visual aids (e.g., icon arrays, bar charts, and line plots), when visual aids are provided instead of rather than in addition to numerical information, and when visual aids differ in iconicity (i.e., when they are more or less abstract). In accord with the growing body of research, we predict that simple, well-designed visual aids will show substantial benefits in many situations, especially when communicating with less numerate individuals.

## Author Contributions

All authors listed on the manuscript have contributed sufficiently to the project to be included as authors. All authors conceptualized the study, obtained funding, and wrote the paper. All authors approved the final version of the manuscript.

### Conflict of Interest Statement

The authors declare that the research was conducted in the absence of any commercial or financial relationships that could be construed as a potential conflict of interest.
